# Genomic selection and genetic gain for nut yield in an Australian macadamia breeding population

**DOI:** 10.1186/s12864-021-07694-z

**Published:** 2021-05-20

**Authors:** Katie M. OConnor, Ben J. Hayes, Craig M. Hardner, Mobashwer Alam, Robert J. Henry, Bruce L. Topp

**Affiliations:** 1 Queensland Department of Agriculture and Fisheries, Maroochy Research Facility, 47 Mayers Road, Nambour, QLD 4560 Australia; 2 Queensland Alliance for Agriculture and Food Innovation, University of Queensland, Maroochy Research Facility, 47 Mayers Road, Nambour, QLD 4560 Australia; 3 Queensland Alliance for Agriculture and Food Innovation, University of Queensland, St Lucia, QLD 4072 Australia

**Keywords:** Horticulture, Plant breeding, Genome-based prediction, Phenotype, Fruit tree

## Abstract

**Background:**

Improving yield prediction and selection efficiency is critical for tree breeding. This is vital for macadamia trees with the time from crossing to production of new cultivars being almost a quarter of a century. Genomic selection (GS) is a useful tool in plant breeding, particularly with perennial trees, contributing to an increased rate of genetic gain and reducing the length of the breeding cycle. We investigated the potential of using GS methods to increase genetic gain and accelerate selection efficiency in the Australian macadamia breeding program with comparison to traditional breeding methods. This study evaluated the prediction accuracy of GS in a macadamia breeding population of 295 full-sib progeny from 32 families (29 parents, reciprocals combined), along with a subset of parents. Historical yield data for tree ages 5 to 8years were used in the study, along with a set of 4113 SNP markers. The traits of focus were average nut yield from tree ages 5 to 8years and yield stability, measured as the standard deviation of yield over these 4 years. GBLUP GS models were used to obtain genomic estimated breeding values for each genotype, with a five-fold cross-validation method and two techniques: prediction across related populations and prediction across unrelated populations.

**Results:**

Narrow-sense heritability of yield and yield stability was low (h^2^=0.30 and 0.04, respectively). Prediction accuracy for yield was 0.57 for predictions across related populations and 0.14 when predicted across unrelated populations. Accuracy of prediction of yield stability was high (r=0.79) for predictions across related populations. Predicted genetic gain of yield using GS in related populations was 474g/year, more than double that of traditional breeding methods (226g/year), due to the halving of generation length from 8 to 4years.

**Conclusions:**

The results of this study indicate that the incorporation of GS for yield into the Australian macadamia breeding program may accelerate genetic gain due to reduction in generation length, though the cost of genotyping appears to be a constraint at present.

**Supplementary Information:**

The online version contains supplementary material available at 10.1186/s12864-021-07694-z.

## Background

Nut yield is the most economically important selection trait of macadamia [1]. In 2017, the Australian industrythe worlds largestproduced a crop of 46,000t of nut-in-shell [2]. Although nut yield is the main trait of focus when selecting new macadamia varieties, it is expensive and difficult to assess in breeding. Nuts are comprised of an outer pericarp green husk, a hard shell testa, and an internal edible kernel. The husk either abscises from the tree along with the nut-in-shell (NIS), or dehisces (splits along a single suture) and the NIS falls to the ground [1]. After harvest, nuts are dehusked mechanically. Yield measurements are usually expressed as NIS or kernel yield per tree [1]. Yield is a complex trait affected by many processes and environmental influences, and is likely controlled by many genes [3, 4]. Previous estimates of yield heritability in macadamia are low (<0.20) [5], indicating that yield is highly likely to be controlled by many loci of small effect. As such, selection for high yield is often made difficult by environmental and genotype x environment interaction (G x E) effects [6]. G x E has been previously documented in macadamia yield [5, 7], though this appeared to be due to a particular characteristic at a particular location, and no work has yet been conducted to understand the repeatable factors behind G x E for yield.

In addition to increased yield, precocious cultivarsthose that produce nuts at an early agemay be commercially attractive due to cash flow at an early age. However, it is not yet known how precocity might affect the rate at which yield begins to plateau in macadamia varieties. In coffee and apple, early-yielding varieties are desirable, particularly those with stable yields over time [8, 9]. For perennial horticulture crops like macadamia, yield stability may be defined as the consistency of yield of individual trees across consecutive years [10]. Unstable yields, due to alternate bearing, is common in some perennial fruit crops and is undesirable as regular income is vital for growers [9, 10]. Research regarding genetic architecture surrounding consistency of yield over years has been limited outside of biennial bearing in apple (e.g. 11, 12). Yield stability is considered an important trait in macadamia by industry [1]. Some macadamia growers report biennial bearing in certain cultivars, such as H2 and 344, which can be problematic.

Selection of new macadamia varieties involves two stages: thousands of seedlings are produced by cross-pollination to create diversity and are assessed in an unreplicated seedling progeny trial (SPT) (sometimes across multiple sites due to space restrictions), then the best performing trees are clonally propagated and evaluated in replicated trials across multiple environments in a candidate cultivar regional variety trial (RVT) [11]. Trees begin to flower and bear fruit around 4 to 5 years after planting, and yield is evaluated for at least another 4 years [5]. Due to the crops long juvenile stage and the need to assess yield over several years to increase the accuracy of predicting performance, traditional breeding has a selection cycle of almost a quarter of a century (22years) [1, 12, 13]. Candidates are then selected for commercial release using a selection index including traits such as yield, kernel recovery (the ratio of kernel to NIS weight; KR), precocity and tree size [12]. Alternative selection strategies are sought to shorten the selection cycle and increase genetic gain.

Genomic selection is a form of marker-assisted selection (MAS) that utilises genome-wide markers to predict genomic estimated breeding values (GEBVs) of individuals, after which the best performers are selected, possibly without phenotyping candidates [14, 15]. As GEBVs can be predicted for individuals at the seedling stage, early selection for elite individuals is possible, thus greatly reducing the selection cycle [15, 16]. GS uses a training or reference population of individuals with known genotypes and phenotypes to construct a model of each markers effect on the trait. To estimate accuracy of prediction, the model is then applied to predict the GEBV of individuals in a validation population, for which measured phenotypes are available. The accuracy of prediction is determined by the correlation between GEBVs and phenotypic observations as a proxy for the unknown true genetic values. MAS can also be conducted using genetic markers of large effect detected through genome-wide association studies (GWAS). GWAS has been conducted in macadamia for nut and kernel traits, but not for yield [17, 18].

Genomic selection was first used in dairy cattle and is being increasingly used to improve genetic gain in both animal and plant breeding programs. With the potential to shorten breeding cycles, long-lived species with slow maturation times may have the most to gain from MAS and GS [19, 20]. Grattapaglia [21] and Lin, Hayes [22] have extensive reviews on the use of GS in forestry and annual species, respectively. The main attraction of GS for perennial crops may be that it can accelerate breeding cycles, thereby increasing the gain per unit time and reducing field trial costs [3, 23, 24]. Sweet cherry [25], peach [26], oil palm [27,29], citrus [30], apple [31, 32], and pear [33, 34] researchers have evaluated the use of GS to increase genetic gain in their breeding programs. A recent study in Japanese chestnut [35] achieved high prediction accuracies for harvest date (r=0.84) and insect infestation (0.60), though yield was not studied.

High prediction accuracy of GS models will improve confidence in selecting elite candidates. Prediction accuracy depends on many factors, including the model, crop, size of the reference population, extent of linkage disequilibrium (LD), marker set, and heritability of the trait of interest [36]. Genetic markers should be in high LD with the genes controlling the trait, in order to capture the genetic variance [14, 37, 38]. In a simulation using animal data, Calus et al. [39] suggested that models using marker densities of LD r^2^=0.2 (average distance of 0.128cM between markers) were superior to those at lower densities. Accurate phenotyping of a large training population, preferably over multiple environments and years (allowing for the study of multiple seasons and tree ages), is required for perennial crops to derive accurate predictions due to the interactions between these factors [23, 40,42].

Recently, an updated version of the *M. integrifolia* genome (v2) was published with 4098 scaffolds anchored to 14 pseudo-chromosomes (745Mb, N50 413kb) [43]. In a study using 4113 SNP markers, of which 90% mapped to v2 genome scaffolds, OConnor, Kilian [44] found that LD decayed rapidly over short distances of the genome. Here, using these same 4113 SNP markers, we explore the potential of GS in macadamia breeding, examining the contribution to genetic gains relative to phenotypic- and pedigree-based selection due to a substantial reduction in generation length. This study aimed to: (i) determine the prediction accuracy of GBLUP (genomic best linear unbiased prediction) methods in predicting GEBVs for nut yield and yield stability across years; (ii) estimate genetic gain using GS strategies compared with traditional breeding methods; and (iii) discuss potential strategies in which GS can be employed to increase genetic gain in macadamia breeding programs. This research is the first study to utilise molecular marker technology for GS in macadamia and, to our knowledge, the first to use GS to predict yield stability over consecutive years for a fruit or nut tree crop.

## Results

### Heritability and accuracy of prediction models

Narrow-sense heritability for yield in the study population was 0.300.08. For yield stability across 4 years, heritability was close to zero (Table1). Variance components, from which estimates of heritability were based, are included in the Supplementary Materials (Supplementary Tables1 and 2).

**Table 1:** Narrow-sense heritability (h^2^) and standard errors (SE) for yield and yield stability

Trait	h^**2**^	SE
Yield	0.30	0.08
Yield stability	0.04	0.04

Moderate prediction accuracy was achieved for yield from cross-validation (CV) using randomly masked individuals (prediction across related populations; 0.570.11). In comparison, yield prediction accuracy was not significantly different from zero for prediction across unrelated populations where families were grouped (0.140.14; Table2). Boxplots showing the observed relationship distributions from the GRM used in predictions are included in the Supplementary Materials (Supplementary Figs.1 and 2).

**Table 2:** Predictive ability and prediction accuracy for yield for each of the five cross-validation (CV) sets for random and family groupings of individuals, and mean and standard error (SE) for each grouping

Grouping	CV set	Predictive Ability	Prediction Accuracy (r)
**Random**	1	0.36	0.66
	2	0.43	0.79
	3	0.30	0.56
	4	0.08	0.16
	5	0.39	0.71
	**Mean**		0.57**
	**SE**		0.11
**Family**	1	0.16	0.29
	2	0.15	0.28
	3	0.03	0.05
	4	0.28	0.51
	5	0.15	0.27
	**Mean**		0.14^NS^
	**SE**		0.14

Results of t-test: ** *p*<0.01, NS indicates not significantly different from zero

For yield stability, high prediction accuracy was achieved for randomly-grouped individuals (0.790.23, *p*<0.01). However, when families were grouped, prediction accuracy was not significantly different from zero (0.280.18; Table3).

**Table 3:** Predictive ability and prediction accuracy for yield stability for each of the five cross-validation (CV) sets for random and family groupings of individuals, and mean and standard error (SE) for each grouping

Grouping	CV set	Predictive Ability	Prediction Accuracy (r)
**Random**	1	0.17	0.86
	2	0.08	0.38
	3	0.29	1.46
	4	0.03	0.15
	5	0.22	1.11
	**Mean**		0.79*
	**SE**		0.23
**Family**	1	0.09	0.43
	2	0.12	0.60
	3	0.06	0.30
	4	0.09	0.43
	5	0.10	0.50
	**Mean**		0.28 ^NS^
	**SE**		0.18

Results of t-test: * *p*<0.05, NS indicates not significantly different from zero

### Comparison of breeding strategies and genetic gain

Two breeding strategies were compared to demonstrate how implementing GS could decrease the breeding cycle and subsequently increase genetic gain (Table4). The number of trees involved in each stage and specific costs are excluded (given uncertainties of, and constantly evolving, genotyping costs).

1. Traditional breeding: Progeny are evaluated in a SPT for at least 8 years to select individuals with elite clonal values for yield and other economically important traits (such as KR, precocity, and tree size) using a selection index. SPT is then followed by a RVT for at least 8 years, where selected elites are clonally propagated and evaluated for more economically important traits across multiple environments.
2. Genomic selection: After germination, the first leaves of each progeny seedling are genotyped for a large number of markers. Genomic prediction is then used to predict GEBVs for yield (and other traits, not shown here). Elite candidates are selected, using a weighted selection index for multiple traits, for establishment and evaluation in the RVT.

**Table 4:** Activities involved in a traditional breeding strategy compared with a simple example of how genomic selection (GS) could be employed in a breeding program. The number of years involved in each activity for the two strategies is shown. Information for traditional breeding is adapted from Topp, Hardner [13]. RVT, regional variety trial; SPT, seedling progeny trial

Year	Traditional breeding	Genomic selection
1	Cross parents, grow seedlings	Cross parents, grow seedlings
2	Age 1: Plant SPT	Genotype, select seedlings using GS
3	Age 2: Trial maintenance	Propagate RVT
4	Age 3: Trial maintenance	Age 1: Plant RVT
5	Age 4: Field evaluations	Age 2: Trial maintenance
6	Age 5: Field evaluations	Age 3: Trial maintenance
7	Age 6: Field evaluations	Age 4: Field evaluations
8	Age 7: Field evaluations	Age 5: Field evaluations
9	Age 8: Field evaluations, select seedlings	Age 6: Field evaluations
10	Propagate RVT	Age 7: Field evaluations
11	Age 1: Plant RVT	Age 8: Field evaluations
12	Age 2: Trial maintenance	Age 9: Field evaluations
13	Age 3: Trial maintenance	Age 10: Field evaluations
14	Age 4: Field evaluations	Release
15	Age 5: Field evaluations	
16	Age 6: Field evaluations	
17	Age 7: Field evaluations	
18	Age 8: Field evaluations	
19	Age 9: Field evaluations	
20	Age 10: Field evaluations	
21	Release	

Here, we consider the generation length (years; L) as the time taken to select individuals to use as parents to produce the next generation of seedlings. Generation length for traditional breeding was 8 (Table 4), as elite individuals are identified after evaluations from age 5 to 8 and are then used as parents for the next generation [1]. By comparison, for strategies employing GS, L was 4. This difference from 8 to 4years is because elite individuals may be identified from genetic markers at a very early age, but cannot be used as parents until reproductive maturity around the age of 4 [1, 45]. The strategy using GS has a much shorter selection cycle (14years) than traditional breeding (21years; Table 1), because it negates the SPT altogether. Both strategies employ RVTs, as it is vital to test the performance of candidate cultivars across multiple environments before commercial release.

For traditional breeding methods, r was calculated as the square-root of yield heritability (0.30=0.55; Table5). The genetic standard deviation of PBLUPs (phenotypic best linear unbiased prediction; using unstandardised yield data) was 1237g. Genetic gain using traditional breeding methods was estimated as 226g/year for 1% selection intensity. At 2.5% selection intensity, genetic gain was reduced to 197g/year. The shorter generation cycle of GS strategies compared with traditional breeding influenced estimates of genetic gain. Genetic gain for GS in related families (randomly-grouped individuals) was more than double that of traditional breeding, at 474g/year for s%=1 and 416g/year for s%=2.5. However, for unrelated population predictions (individuals grouped by family), traditional breeding achieved higher genetic gain than GS, which was estimated to be 119g/year for s%=1 and 105g/year for s%=2.5.

**Table 5:** Genetic gain of yield (G, in g/year) for traditional breeding and genomic selection methods as outlined in Table 4. Genetic gain was calculated using Eq. 6, where i was a function of the percentage of the population selected (s%) as given by Falconer and Mackay [46], r is the square-root of yield heritability for traditional breeding or the prediction accuracy of genomic selection model, is the standard deviation of PBLUPs (in g), and L is the generation length (in years)

Method	s%	i	r	(g)	L (years)	G (g/year)
**Traditional breeding**	1	2.665	0.55	1237	8	226
	2.5	2.338	0.55	1237	8	197
**Genomic selection**						
**Randomly-grouped (related population)**	1	2.665	0.57	1237	4	474
	2.5	2.338	0.57	1237	4	416
**Family-grouped (unrelated population)**	1	2.665	0.14	1237	4	119
	2.5	2.338	0.14	1237	4	105

## Discussion

### Comparison of prediction models and cross-validation methods

This study is the first to investigate the use of genomic prediction to improve genetic gain for yield and yield stability in macadamia breeding. Our results suggest that yield-based traits are complex and highly polygenic, as indicated by low heritability, and that GS offers a suitable method to select genotypes to improve yield. Prediction accuracy is strongly influenced by the relatedness between training and validation populations [15], and unrelated population predictions are expected to perform poorly compared to related family prediction [47]. This pattern was observed across the models in the current study; model prediction accuracy for randomly-grouped individuals was higher than family-grouped individuals (predictions in unrelated populations). This is because with random groupings for CV, the training set includes full-sibs from the validation set (e.g. progeny from the same cross will be split across the training and validation sets), and so large blocks of chromosomes will be shared between the training and validation sets. The low to moderate prediction accuracies observed by Muranty, Troggio [31] in apple were attributed to predictions across unrelated populations. By comparison, Kumar, Chagne [32] achieved high prediction accuracies (0.70 to 0.90) for apple fruit quality traits, with individuals randomly allocated to CV groups.

The CV method of family-grouped prediction represents an extreme version of the potential real-world application of GS in macadamia where predictions are performed across unrelated populations. It is likely that the training and target populations will actually be more closely related as there is often an overlap of cultivars used as parents between breeding populations, and elite individuals from one population are commonly used as parental germplasm in subsequent generations [13]. It is expected that prediction of GEBVs in a breeding program will, therefore, have accuracies closer to that of the randomly-grouped predictions compared with unrelated population predictions presented in this study. Employing GS in a population closely related to that on which the model is based would provide more accurate predictions of yield. However, more research is needed using large training population sizes with validation sets of whole family groups to improve prediction accuracy before GS can be applied in macadamia breeding.

The implementation of GS in macadamia may include prediction and deployment across environments. The current study population had limited replication of genotypes across environments and did not include G x E interactions in prediction models as preliminary results found no evidence of G x E in this experimental material [48]. Previous studies have found some evidence that G x E may affect macadamia yield [5, 12]. However, research has not yet identified any repeatable factors than can be used for targeted deployment.

### Factors affecting accuracy of genomic prediction

The prediction accuracy for yield in the current study was moderate for randomly-grouped individuals (r=0.57), and comparable to the prediction accuracy of yield as measured by phenotypes (h^2^=0.30, h=r=0.55). These similar values for r demonstrate that the genomic prediction accuracy estimated in the current study will provide similar gain as phenotypic analysis, regardless of the time advantage in GS strategies. The prediction accuracy achieved in GS in this study was not as high as reported in some other horticulture crops, which may be attributed to several factors. Estimates of macadamia yield in the current and previous studies [5] involve a large non-genetic component, as indicated by the low heritability and/or high non-additive genetic variation for this trait, and suggest a quantitative nature of inheritance. Yield measurement inaccuracies can occur when overlapping canopies result in a mixture of dropped nuts from neighbouring trees. Additionally, the method used to obtain DNIS (dry nut-in-shell) weight per harvest assumes that the moisture content of the 1kg sample is consistent through the entire harvest. For these reasons, measuring macadamia yield is very different to measuring yield in other fruit crops, which may inhibit accurate yield prediction.

This study is, to our knowledge, the first to estimate heritability of stability of yield over consecutive years for a nut tree, and use genomic prediction to predict genetic values of yield stability. Biennial bearing in apple has been researched by multiple authors. Guitton, Kelner [49] found three QTLs associated with biennial bearing that explain 50% of phenotypic variability. Additionally, Durand, Guitton [50] suggested that irregular bearing in apple may be more influenced by factors affecting floral induction rather than those affecting fruit set or drop. Predictions using randomly-grouped individuals were moderately high for yield stability, though this may be due to the low heritability of the trait inflating prediction accuracy. The low heritability of yield stability indicates that yield fluctuations between years is very weakly controlled by genetics, and may be more influenced by non-genetic factors. Thus, it would be up to breeders to determine the value of including yield stability in a selection index when identifying elite candidates for further testing.

The population size of this study was limited compared to other studies in fruit crops, though it did consist of a large number of full-sib families. In the first study of GS in cross-pollinated fruit crop species, Kumar, Chagne [32] obtained high model accuracy for fruit quality traits in apple. They used a much larger population (1120 seedlings) than the current study, albeit from a smaller parent population (seven full-sib families from four female and two male parents), and prediction accuracy ranged from r=0.70 to 0.90 using RR-BLUP and Bayesian LASSO methods. GS in citrus achieved high (r>0.7) prediction accuracy for some fruit quality traits using around 800 individuals, with the GBLUP model consistently out-performing other models [30]. Similarly, using a Japanese pear population of 86 parents and 765 progeny, prediction accuracy varied between models and CV methods, and was commonly greater than 0.5 [33]. However, the correlations found for citrus and Japanese pear may be inflated, since negative correlation coefficients were set to zero when calculating prediction accuracy for these studies. Increasing the size of a phenotyped and genotyped training population would increase the accuracy of yield prediction in macadamia.

LD between markers and genes controlling target traits is essential for GS [15]. Previous studies have suggested increasing the number of markers used in GS may not necessarily achieve better accuracies. Studies investigating the prediction accuracy of GS in citrus, Japanese pear and apple all used fewer SNP markers than the current study (1841, 1502 and 2500, respectively) [30, 32, 33]. Using the same 4113 SNP markers used in the current study, OConnor, Kilian [44] found that SNPs within 1kb distance of each other on a scaffold (*M. integrifolia* v2 genome assembly, 4098 scaffolds) had an average LD of r^2^=0.124, with LD decaying rapidly over short distances and more moderately over long distances [44]. These results are important for the current study to determine that genetic markers capture genetic variance of the target trait [15, 38]. Increasing the density of markers across the genome could lead to increased prediction accuracies, as suggested by Calus, Meuwissen [39], where models with r^2^=0.2 between markers were more accurate than models with fewer markers and lower densities. Future analysis of LD in macadamia could employ the use of an updated macadamia reference genome (45) to determine the distribution of markers across chromosomes, and include corrections for population structure and cryptic relatedness.

Genetic recombination occurs with successive generations of breeding, which may affect the linkage between markers and genes controlling target traits [51]. Furthermore, selection for improved individuals will also alter the frequency of alleles in the population [52]. These changes over generations will have consequences for genomic prediction accuracy. Meuwissen, Hayes [15] estimated that the prediction accuracy of GS models will decrease at around 5% per generation, due to recombination. Thus, it is necessary to recalibrate the model after every few generations as genetic variance explained by the markers will change, along with the allelic frequencies in the population [40, 53]. To aid in model recalibration, Grattapaglia [21] suggested that selection candidates should remain in the field and be grown for 5 to 6 years to provide phenotypes for updating the model. This strategy could be employed in macadamia to ensure accuracy of predictions through subsequent generations of GS.

### Genetic gain from genomic selection

The results of this study indicated that genetic gain in macadamia breeding was particularly influenced by the length of the breeding cycle. Genotyping seedlings at a very early age, for example using their first leaf after germination, to identify high-yielding individuals through GS could halve the length of the SPT. Subsequently, elite trees could be cross-pollinated to produce the next generation as soon as they begin to flower, which is usually around the age of four. From there, clonally replicated trees could be phenotyped for other economically important traits, and candidate cultivars identified using a selection index. Similarly in apples, Muranty, Troggio [31] suggested that GS could increase genetic gain per year compared with conventional breeding, by shortening the breeding cycle from 7 to 4 years. In contrast, prediction accuracy was not sufficient for all target traits in oil palm to reduce the generation interval, meaning that breeding would still require the testing of progeny [54]. The authors suggested that if given the resources to increase the size of the training set, and a greater ability to model G x E interactions, GS could be a valid option to increase genetic gain in oil palm.

In their review of GS in apple by Kumar et al. [32, 55], van Nocker and Gardiner [56] proposed using MAS and GS to identify elite apple accessions and then, to decrease time to reproductive maturity, to implement a regime to promote early flowering. Fruit would be phenotyped over two early seasons, and then BVs compared with the predicted GEBVs to analyse genetic gain. Using these methods, candidate cultivars could be clonally propagated 7 years earlier than traditional breeding. However, the predicted beneficial outcomes of using GS in apple may not be as achievable if predictions were to occur across families rather than in randomly-grouped individuals, as has been shown here in macadamia.

### Logistics of using genomic selection to increase genetic gain

The opportunity to employ GS in a wider range of crops is increasing with declining genotyping costs and advancements in technology [20, 51, 57]. Implementing genomics-assisted breeding may be expensive due to the cost of genotyping large numbers of candidates at each cycle. However, the cost of genotyping will be a trade-off with a decrease in the costs needed for phenotyping [58] due to the elimination of costs involved in measuring yield during the SPT. An evaluation of costs involved in MAS versus GS has been made for maize and wheat, and GS outperformed MAS even when prediction accuracies were low [58]. Breeders should compare selection strategies to determine which combination of genotyping and phenotyping is most suitable for their crop and program to maximise accuracy of trait prediction in fruit crops [31].

Currently, costs involved in genotyping may restrict the implementation of GS in the Australian macadamia breeding program. To reduce genotyping costs, delaying GS to deploy on a smaller population size may be a viable option, similar to a strategy proposed by Gardiner, Volz [59]; to reduce the size of the seedling population to be genotyped, pre-screen the population for essential traits first. Seedlings could be grown out as per a traditional SPT, but only evaluated to age four, and precocious (early bearing) trees evaluated for KR (high KR attracts a higher commission per kilogram than low KR [60]). Breeders could pre-select precocious seedlings with high KR, genotype this reduced number of potentially elite individuals, and then the highest-yielding trees could be selected through GS for evaluation in RVTs. Longer generation intervals, due to phenotyping for precocity and KR for several years initially, would lead to a lower genetic gain using this strategy than GS of more seedlings at an earlier stage; however, it may be a more cost-effective option. Additionally, whilst implementing GS in macadamia may not decrease the time from seed to reproductive maturity, selecting for precocious individuals may aid in producing more individuals with a shortened juvenile stage. Reaching reproductive maturity at an earlier stage will further increase genetic gain by reducing the generation length of 4 years in the GS strategy. Extending quantitative modelling of different options for using GS in a breeding program may help to compare possible approaches and identify optimum strategies. Comparing costs of traditional breeding versus strategies using GS is not the focus of this study, though this should be evaluated to determine the prospect of implementing GS in the Australian macadamia breeding program.

### Future research using GS in macadamia

Future work employing GS to increase genetic gain in macadamia could investigate other economically important traits, such as tree size. In the same population as the current study, O'Connor et al. [18] found 16 QTLs linked with trunk circumference. The large number of markers associated with this trait, compared with other traits in the study, means that GS may be more appropriate than GWAS and MAS to increase genetic gain, given the seemingly quantitative nature of trunk circumference. GS may also be a good candidate for other traits, such as resistance to diseases and pathogens, including husk spot and phytophthora [61]. Furthermore, the significant associations identified between traits and markers could be incorporated into GS models. Genomic prediction methods including BayesR and BayesB allow the effect of some markers, such as those of significant effect, to be larger than others [15, 62]. Different model types could therefore be tested in the future to determine which are the most accurate in predictions.

Further work could also include multi-trait models to investigate whether the inclusion of additional traits, such as trunk circumference and nut weight, increases the accuracy of yield prediction. Jia and Jannink [63] found that prediction accuracy was increased for a trait with low heritability by including information for a correlated trait with high heritability. Estimates of heritability and genetic correlations between yield and various component traits have been made [48] and, thus, this information could be used to inform multi-trait GS. Distinctions can also be made between linked QTLs (linkage between multiple QTLs affecting different traits) and pleiotropic QTL (one gene affecting multiple traits), using multi-trait methods, like those employed by Bolormaa, Pryce [64].

Finally, future GS analyses could involve more genetic markers and/or more evenly-distributed markers across the genome. This approach may ensure that small-effect loci are captured, since LD in macadamia decays rapidly over short distances [44]. With the aid of the recently published macadamia reference genome (45), future sequencing of individuals for GS analysis and the calling of SNPs may be more accurate and avoid potential issues associated with allelic dropouts [44].

## Conclusions

We found that genomic prediction accuracy of nut yield in macadamia in randomly-grouped individuals was moderate, at r=0.57, and similar to the accuracy of traditional breeding. Prediction accuracy across unrelated populations (r=0.14) was lower than for prediction across randomly-grouped individuals. However, due to the relatedness in parental germplasm between subsequent breeding generations, a realistic prediction accuracy would likely be similar to that of randomly-grouped rather than family-grouped individuals. Additionally, we believe that this study is the first to estimate heritability of yield stability across years for a nut tree, as well as genomic prediction for this trait. Genetic gain for yield using GS (474g/year) was more than double that of traditional breeding methods (226g/year), largely due to the generation length being halved in GS. Our results indicate that GS is a viable option to increase genetic gain of yield in macadamia, though validation in a separate population is required. Further research and validation into the use of marker-assisted selection for other key traits in the seedling stage, including kernel recovery and tree size, are needed. Additionally, increasing the number and spread of markers across the genome may capture more causal polymorphisms through LD and lead to higher prediction accuracy.

## Methods

### Plant material and phenotyping

This study involves a subset of individuals from a progeny population in the Australian industry macadamia breeding program, from which seedlings were produced, established and assessed in field trials by Horticulture Australia Limited, CSIRO and the Queensland Government. The entire progeny population consisted of approximately 2000 seedlings across 141 full-sib families from crosses between 47 publicly available parents, with 136 progeny per family (mean 14) [7, 11]. Trees were planted across nine sites in south-eastern Queensland and north-eastern New South Wales, Australia, between 2001 and 2003 in single tree plots in incomplete block design with replication of families [11]. Trees were planted at 4m distances within rows, and 8m between rows.

As described by OConnor, Kilian [44], 295 unreplicated macadamia seedlings from 32 full-sib families (reciprocals combined) from crosses between 29 parents (711 progeny per family) across four sites were chosen. All families had at least one parent in common with another family. Progeny within families were selected to achieve an approximately equal number of low- and high-yielding individuals per family, based on breeding values for cumulative nut-in-shell yield to age 8years after planting (data not shown), to increase power. Trial sites were within the commercial production zone for macadamia and included Hinkler Park (HP) and Alloway (AL) near Bundaberg, Queensland, and East Gympie (EG) and Amamoor (AM) near Gympie, Queensland. Clones of five parental genotypes were planted at each of the four sites, with a further 13 parental clones planted at AL. Eleven of the 29 parents were not included in the study as they were not present at the study sites. Sites were planted in 2001 (EG, HP), 2002 (AL, part of AM), and 2003 (part of AM).

Historical data were used in this study. Yield was evaluated on an individual tree basis from ages 5 to 8 after planting. Each year, nuts-in-husk were manually harvested from the ground in multiple harvests from February to August. A final strip harvest was undertaken at the end of the season, in which the nuts remaining in the tree were removed with poles and hooks. Nuts were dehusked mechanically and weighed to obtain a wet nut-in-shell weight. For each tree, a 1kg sample was taken (where available) and dried to approximately 1.5% moisture content at 35C for 48h, 45C for 48h, and 55C for 48h, based on protocol by Prichavudhi and Yamamoto [65]. Samples were then weighed to obtain a dry nut-in-shell (DNIS) weight, with the moisture content used to calculate a total DNIS weight per harvest. DNIS weights were summed across harvests to obtain the total NIS yield per tree each year. For some subsequent analyses, individual tree yield data for each age (age 5 to 8) were standardised by dividing the observation by the standard deviation of each site and age to reduce the bias in genetic values due to heterogeneity of variance among trial sites (following Hardner [7]).

### Phenotypic data analysis

An individual-tree linear mixed model was used to predict individual tree effects (phenotypic BLUPs; PBLUPs) using yield data across the four years (ages 5 to 8):

1 $$ Yield= Site+ Block+ Type+ Number\ Neighbours+ Age+ Year+ Tree+ error $$

where **Yield** was the standardised yield of an individual tree in 1 year; **Site** was the fixed effect of the location of the tree (AM, AL, EG and HP in Queensland); **Block** was the fixed effect of planting block within a Site; **Type** was the fixed effect of method used to propagate the tree (seedling progeny or clonally propagated parent); **Number Neighbours** was the fixed effect of the number of trees on either side of that tree within the planting row, to allow for influence on phenotype of gaps created by the death of neighbouring trees; **Age** was the fixed effect of age after planting of the tree; **Year** was the fixed effect of calendar year that yield was harvested (as trees were planted in different years across the sites); **Tree** was the random effect of individual tree (with no replication of genotypes) within Site, without any pedigree or genetic relationship information, and **error** was a vector of random deviations e~N (0, ${\boldsymbol{\sigma}}_e^{\mathbf{2}}$) where ${\boldsymbol{\sigma}}_e^{\mathbf{2}}$ is the error variance. As multiple years of yield data were used in the model, a single prediction of mean yield across years for each tree was predicted. PBLUPs were also obtained for trees in each of the four ages (ages 5 to 8) as per Eq. 1, without the effects of Age and Year. Yield stability of each tree was quantified as the standard deviation (SD) of the four annual PBLUPs predicted from the above model.

### SNP genotyping and imputation

This study used genetic markers obtained as described by OConnor, Kilian [44] and briefly outlined here. DNA was extracted from leaves of 295 seedlings and their parents, and sequenced by Diversity Arrays Technology (DArT) Pty Ltd. DArT performed digestion/ligation reactions using a combination of *Pst*I and *Hha*I restriction enzymes and barcoded adaptors. After PCR, samples were pooled, applied to c-Bot (Illumina) bridge PCR, and then sequenced using Illumina Hiseq2500 for 77cycles. Sequences were processed using proprietary DArT pipelines, with SNP markers detected based on parsing sequence clusters. Missing calls were imputed using the probabilistic principal components analysis (PPCA) method [66] with 97.2% accuracy, which was determined by excluding an additional 10% of missing values and calculating the correlation between the imputed calls and the original dataset. Quality control was performed using pre-imputation parameters, including 50% original call rate, 2.5% minor allele frequency, and a test of Mendelian inconsistencies (parent-offspring trio opposing homozygotes) determined using 16 (50%) of the families. This quality control resulted in 4113 SNPs for genomic analysis.

### Genomic BLUP models

An additive genomic relationship matrix (GRM) was constructed among all individuals using the 4113 SNPs, as per VanRaden [67] and detailed in OConnor, Kilian [44]. GBLUP models were used to estimate GEBVs for each tree using ASReml-R [68]. Preliminary analyses indicated no significant difference in prediction accuracy between additive genomic effects and sites [48], and thus G x E was not included in analyses.

2 $$ Yield= Site+ Block+ Type+ Number\ Neighbours+ Age+ Year+ Accession+ SiteTree+ error $$

where **Accession** is the tree effect modelled as the additive genetic effect of the individual, assumed random ~ N (0,**G** ${\boldsymbol{\sigma}}_{\boldsymbol{g}}^{\mathbf{2}}$), where **G** is the GRM, modelled from SNP effects (where 0, 1, and 2 represents the dosage of the reference allele), and ${\boldsymbol{\sigma}}_{\boldsymbol{g}}^{\mathbf{2}}$ is the additive genetic variance captured by the SNP markers; and **SiteTree** is the permanent environment random effect of each tree within site across years, uncorrelated among trees, assumed random ~ N (0, ${\boldsymbol{\sigma}}_{\boldsymbol{pe}}^{\mathbf{2}}$) where ${\boldsymbol{\sigma}}_{\boldsymbol{pe}}^{\mathbf{2}}$ is the variance attributed to a permanent environment effect. As multiple years of yield data were used in the model, a single prediction for each accession for mean yield GEBV across years was obtained.

To determine the accuracy of genomic prediction for yield stability over multiple years, GEBVs were obtained using the following model:

3 $$ Yield\  SD= mean+ Accession+ error $$

where **Yield SD** is the standard deviation of PBLUPs for ages 5 to 8 across all sites.

An estimate of genomic narrow-sense genomic heritability (h^2^) of average yield was made based on the variance components of Eq. 2 (Supplementary Table2) using the pin function in R [69]:

4 $$ {h}^2=\frac{\sigma_g^2}{\sigma_g^2+{\sigma}_{pe}^2+\raisebox{1ex}{$\ {\sigma}_e^2$}\!\left/ \!\raisebox{-1ex}{$4$}\right.} $$

where ${\sigma}_g^2$ is the additive genetic variance, ${\sigma}_{pe}^2$ is the variance attributed to a permanent environment effect, and ${\sigma}_e^2$ is the residual variance (divided by 4 for the 4 years of data). For yield stability, an estimate of h^2^ was calculated as ${\sigma}_g^2$ / (${\sigma}_g^2+{\sigma}_e^2\operatorname{}$) from variance components of Eq. 3.

### Model validation

The prediction accuracies of the GEBVs from the models above were determined using five-fold cross-validation (CV). In turn, 20% of phenotypes were masked (set to missing) in a validation set, and data for the remaining 80% of individuals were used as a training set to train the model and predict the masked values. This process was repeated five times until all subsets were used in the validation set, with each individual used only once in the validation set.

Individuals were assigned to one of five groups for the five-fold CV using two grouping techniques for predictions: random and related family groups. For the random CV, individuals were selected for the training and validation group at random (randomly-grouped). Here, full-sibs were randomly allocated across the training and validation groups, and so predictions were performed on individuals that were related to the training population. For the second method, individuals were grouped by family and related families (those with common parents) and grafted parents (family-grouped), to give approximately equal-sized groups. Thus, entire full-sib families were either in the reference set or in the validation set, and predictions were performed on families unrelated to the training population (unrelated population). This second method represents a more extreme version of the application of GS, where the two populations are not closely related.

Prediction accuracy (r) for each of the five CVs was calculated as:

5 $$ r=\frac{corr\left( GEBVs, PBLUPs\right)}{\surd {h}^2} $$

where the PBLUPs were estimated from Eq. 1, and heritability (h^2^) was estimated from Eq. 4. The correlation between GEBVs and PBLUPs is predictive ability. Mean prediction accuracies and standard errors were calculated across the five CVs, and t-tests were performed to determine if prediction accuracies were significantly different from zero.

### Comparison of breeding strategies and genetic gain

A simple comparison of breeding strategies was made to demonstrate how GS could be effectively incorporated into the macadamia breeding program to reduce selection time and increase genetic gain. Genetic gain (G, grams/year) was calculated for yield for traditional breeding and GS methods using the following equation derived from Falconer [52]:

6 $$ \Delta  \mathrm{G}=\frac{i\times r\times \sigma }{L} $$

where *i* is selection intensity as a function of the proportion of the population selected, *r* is square-root of yield heritability for traditional breeding or the prediction accuracy of the GS model, and *L* is generation length in years. Here, is the genetic standard deviation, which was calculated as the standard deviation of PBLUPs from Eq. 1, but using unstandardised yield data to give a value in grams. In traditional breeding, approximately 2000 seedlings are evaluated and 1% (20/2000) of the SPT population are further tested in an RVT [11]. Here, the selected percentage of the population (%s) has been increased from 1 to 2.5% in an attempt to reduce the probability of not selecting truly elite germplasm under GS. As such, in this equation, *i*=2.665 and 2.338, for 1 and 2.5% selected, respectively, as taken from Falconer and Mackay [46]. We assume that genetic gain for RVT selection is the same across selection strategies, and so genetic gain is only calculated here for the SPT.

## Supplementary Information

**Additional file 1: Supplementary Table1.** Variance components for phenotypic BLUPs derived from Eq. 1. **Supplementary Table2.** Variance components for genomic BLUPs derived from Eq. 2. **Supplementary Figure 1.** Boxplots showing the observed relationship distributions from the GRM for: full siblings, half siblings, parent offspring, parent-parent, and unrelated relationship groups. **Supplementary Figure 2.** Boxplot showing the observed relationship distributions from the GRM for the diagonals (identity).

## Data Availability

The datasets generated and/or analysed during the current study are available from The University of Queenslands Institutional Data Access/Ethics Committee, but restrictions apply to the availability of these data. Data are however available from the authors upon reasonable request and with permission of The University of Queensland for researchers who meet the criteria for access to confidential data. SNP data can be accessed from 10.14264/uql.2018.491 and yield data from 10.14264/5f8abc3.

## Abbreviations

AL: Alloway (study location site)
AM: Amamoor (study location site)
CV: Cross-validation
DArT: Diversity Arrays Technology
DNIS: Dry nut-in-shell
EG: East Gympie (study location site)
GBLUP: Genomic best linear unbiased prediction
GEBV: Genomic estimated breeding value
GRM: Genomic relationship matrix
GS: Genomic selection
GWAS: Genome-wide association study
G x E: Genotype by environment interaction
HP: Hinkler Park (study location site)
KR: Kernel recovery (ratio of kernel to nut-in-shell)
LD: Linkage disequilibrium
MAS: Marker-assisted selection
NIS: Nut-in-shell
PBLUP: Phenotypic best linear unbiased prediction
QTL: Quantitative trait loci
RVT: Regional variety trial
SD: Standard deviation
SE: Standard error
SNP: Single nucleotide polymorphism
SPT: Seedling progeny trial
