# Supplementary material for: Genomic selection and genetic gain for nut yield in an Australian macadamia breeding population
Source: BMC Genomics. 2021 May 20;22:370. doi: 10.1186/s12864-021-07694-z (PMC8139092; doi:10.1186/s12864-021-07694-z)

# Supplementary material

**Supplementary Table 1: Variance components for phenotypic BLUPs derived from equation 1.**

|  | component | std.error | z.ratio | bound | %ch |
| --- | --- | --- | --- | --- | --- |
| Tree!Tree | 0.3986266 | 0.04521415 | 8.816413 | P | 0 |
| units!units | 0.5331006 | 0.02421509 | 22.015219 | P | 0 |

**Supplementary Table 2: Variance components for genomic BLUPs derived from equation 2.**

|  | component | std.error | z.ratio | bound | %ch |
| --- | --- | --- | --- | --- | --- |
| vm(Tree, gainv) | 0.1449804 | 0.05579228 | 2.598574 | P | 0 |
| SiteTree!SiteTree | 0.2138626 | 0.05181182 | 4.127679 | P | 0 |
| units!units | 0.5254956 | 0.02389802 | 21.989083 | P | 0 |

**Supplementary Figure 1: Boxplots showing the observed relationship distributions from the GRM for: full siblings, half siblings, parent offspring, parent-parent, and unrelated relationship groups.**


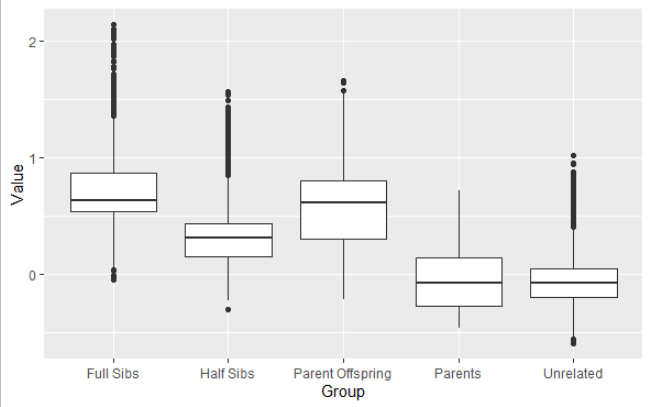


**Supplementary Figure 2: Boxplot showing the observed relationship distributions from the GRM for the diagonals (identity).**


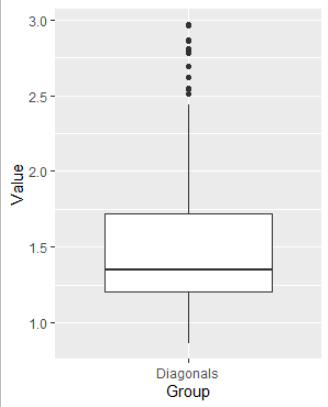

Supplement: Supplementary file 1 — Additional file 1: Supplementary Table1. Variance components for phenotypic BLUPs derived from Eq. 1. Supplementary Table2. Variance components for genomic BLUPs derived from Eq. 2. Supplementary Figure 1. Boxplots showing the observed relationship distributions from the GRM for: full siblings, half siblings, parent offspring, parent-parent, and unrelated relationship groups. Supplementary Figure 2. Boxplot showing the observed relationship distributions from the GRM for the diagonals (identity). [file 12864_2021_7694_MOESM1_ESM.docx]
